# Harmonization of malaria rapid diagnostic tests: best practices in labelling including instructions for use

**DOI:** 10.1186/1475-2875-13-505

**Published:** 2014-12-17

**Authors:** Jan Jacobs, Barbara Barbé, Philippe Gillet, Michael Aidoo, Elisa Serra-Casas, Jan Van Erps, Joelle Daviaud, Sandra Incardona, Jane Cunningham, Theodoor Visser

**Affiliations:** Department of Clinical Sciences, Institute of Tropical Medicine, Antwerp, Belgium; Department of Microbiology and Immunology, University of Leuven, Leuven, KU Belgium; Malaria Branch, Division of Parasitic Diseases and Malaria, Center for Global Health, Centers for Disease Control and Prevention, Atlanta, GA USA; Roll Back Malaria Partnership, Geneva, Switzerland; The Global Fund to Fight AIDS, Tuberculosis and Malaria, Geneva, Switzerland; The Foundation for Innovative New Diagnostics, Geneva, Switzerland; WHO Global Malaria Programme, World Health Organization, Geneva, Switzerland; Clinton Health Access Initiative, Boston, MA USA

**Keywords:** Malaria RDT, Harmonization, Best practices, Labeling, Instructions for use

## Abstract

**Background:**

Rapid diagnostic tests (RDTs) largely account for the scale-up of malaria diagnosis in endemic settings. However, diversity in labelling including the instructions for use (IFU) limits their interchangeability and user-friendliness. Uniform, easy to follow and consistent labelling, aligned with international standards and appropriate for the level of the end user’s education and training, is crucial but a consolidated resource of information regarding best practices for IFU and labelling of RDT devices, packaging and accessories is not available.

**Methods:**

The Roll Back Malaria Partnership (RBM) commissioned the compilation of international standards and regulatory documents and published literature containing specifications and/or recommendations for RDT design, packaging and labelling of *in vitro* diagnostics (IVD) (which includes RDTs), complemented with a questionnaire based survey of RDT manufacturers and implementers. A summary of desirable RDT labelling characteristics was compiled, which was reviewed and discussed during a RBM Stakeholder consultation meeting and subsequently amended and refined by a dedicated task force consisting of country programme implementers, experts in RDT implementation, IVD regulatory experts and manufacturers.

**Results:**

This process led to the development of consensus documents with a list of suggested terms and abbreviations as well as specifications for labelling of box, device packaging, cassettes, buffer bottle and accessories (lancets, alcohol swabs, transfer devices, desiccants). Emphasis was placed on durability (permanent printing or water-resistant labels), legibility (font size, letter type), comprehension (use of symbols) and ease of reference (*e.g.* place of labelling on the box or cassette packaging allowing quick oversight). A generic IFU template was developed, comprising background information, a template for procedure and reading/interpretation, a selection of appropriate references and a symbol key of internationally recognized symbols together with suggestions about appropriate lay-out, style and readability.

**Conclusions:**

The present document together with its additional files compiled proposes best practices in labelling and IFU for malaria RDTs. It is expected that compliance with these best practices will increase harmonization among the different malaria RDT products available on the market and improve their user-friendliness.

**Electronic supplementary material:**

The online version of this article (doi:10.1186/1475-2875-13-505) contains supplementary material, which is available to authorized users.

## Background

### Global malaria burden and importance of diagnostic testing

As the global burden of malaria is decreasing, diagnostic testing for malaria before starting treatment is crucial to avoid overtreatment with anti-malarials and delayed management of non-malaria febrile illnesses. To this end, since 2010, the World Health Organization (WHO) broadened its recommendations for parasitological confirmation of malaria diagnosis before treatment to all age groups [1, 2]. Furthermore, in 2012, the “‘T3: Test. Treat. Track.” initiative called to scale-up diagnostic testing, better target treatment and improve surveillance systems [3].

### Scale-up of malaria diagnosis has mainly been achieved by rolling-out of RDTs

Case management treatment guidelines recommend diagnostic testing by use of quality light microscopy or alternatively by quality rapid diagnostic tests (RDTs) [2]. RDTs are instrument-free test devices (primarily a plastic cassette that encloses a test strip) capable of providing results within 30 minutes. They require minimal training and expertise compared to microscopy and can be performed by less-skilled health workers [4, 5]. In endemic settings, diagnosis of *Plasmodium falciparum* infections made by well-performing RDTs has been shown to be equal or superior to routine microscopy, and appears to be more cost-effective than microscopy [6–10]. RDTs are largely accountable for the scale-up of parasitological diagnosis [1, 5, 11].

### Limitations of RDTs: variety of procedures, limited user-friendliness

Despite their robustness and apparent simplicity, RDTs have their limitations [12]. Surveys suggest there are currently at least 200 RDT products on the market [13, 14], which vary both in type and design of test device (shape of cassette, number of wells), accessories (specimen transfer devices, lancets) and procedure (specimen volume, numbers of buffer drops, reading time). In addition, there are many differences in labelling and terminology for the RDT box, device packaging, buffer bottles and accessories [5]. This diversity contributes to the limitations in terms of user-friendliness especially since RDTs are often performed by less-skilled heath workers. Such differences create challenges in terms of training when operators are required to switch from one RDT product to another. Problems with operating different RDT products are particularly noted during the early phases of RDT introduction at the country level [5].

### Harmonization and enhanced user-friendliness of RDTs

Enhanced harmonization of malaria RDT characteristics is expected to facilitate procurement and supply management as well as to reduce training/re-training and supervision when switching from one RDT product to another or when two or more RDT products are concurrently used in a country. In addition, it is expected to improve general adherence to manufacturer’s recommended procedures and reduce operational errors. Uniform, easy to follow and consistent labelling and instructions for use (IFU) are crucial to a good performance of the test and to harmonization of RDTs [15], but a comprehensive resource containing specific information about best practices in instructions for use, labelling, of RDT devices, packaging and kit accessories is not available.

In July 2012, the Diagnostics Work Stream of the Roll Back Malaria Partnership (RBM) Procurement and Supply Chain Management Working Group commissioned the Institute of Tropical Medicine (ITM, Antwerp, Belgium) to assess the level of similarities and differences between current commercially available malaria RDTs, and to identify opportunities and challenges for enhanced and rational harmonization of malaria RDT characteristics. As a guidance for the latter, regulatory documents (such as legislation, regulatory guidance and standards) and published literature relating to design, packaging and labelling of *in vitro* diagnostics (IVD) were compiled and assessed for their applicability and feasibility to incorporate into malaria RDTs. The process and results of these findings are described here; the results of the comparative assessment of commercially available RDTs and accessories against these findings is presented elsewhere.

## Methods

### Working document of best practices in labelling including instructions for use of RDTs

In a first step, regulatory guidance and guidelines for medical devices were identified and assessed for applicability for malaria RDTs. They included documents issued by the Global Health Task Force (GHTF) (now International Medical Device Regulators Forum (IMDRF)) [15–17], the International Organization of Standardization (ISO norms) [18–20], the European Commission (EC directives) [21–23], the USA Food and Drug Administration (FDA) (http://www.fda.gov/) regulations relating to labelling of IVDs [24] and a the GP42-A6 guidelines (Procedures and devices for the collection of diagnostic capillary blood specimens) from the Clinical and Laboratory Standards Institute (CLSI) [25]. In addition, information about end-user errors in RDTs and readability of instructions was retrieved from scientific literature and complemented with ITM field observations and comments from manufacturers and implementers obtained through face-to-face structured interviews (five manufacturers and 9 implementers) and an internet-based questionnaire (16 implementers).

Based on this review, the ITM team drafted a working document compiling suggestions for consideration towards enhanced harmonization and user-friendliness, addressing issues of labelling and design and construction of RDT cassette and accessories. Interviews with manufacturers, based on these suggestions, were conducted and manufacturers rated the considerations for relevance and feasibility for incorporation into manufacturing processes. Full details of methods and sources can be retrieved on the RBM website [26].

### Consultation meeting with malaria control programme implementers, manufacturers and experts in regulatory affairs

The same ITM working document was extensively discussed during a RBM Stakeholder Consultation meeting (December 3^rd^ – 5^th^ 2013, ITM) through small group and plenary review sessions. The meeting gathered 81 participants representing (i) country programme implementers (laboratory managers from the National Malaria Control Programmes (NMCP), laboratory experts involved in RDT implementation research, n = 27), (ii) experts involved in global support to RDT implementation (n = 16), (iii) experts in IVD regulatory matters (n = 22) and (iv) RDT manufacturer representatives (16 participants from nine companies), involved in research and development, quality assurance, production or business development and with sales estimated to represent over 90% of the current public market in 2010 [27].

The meeting resulted in a consolidated list of suggested specifications promoting harmonization and set out further steps and timelines towards enhanced harmonization of RDT characteristics [28]. To ensure follow-up, the meeting endorsed the RBM Procurement and Supply Chain Management Working Group to create an *ad-hoc* taskforce and participants were invited to express their interest to join this taskforce.

### MRDT harmonization task force (HarT)

The “mRDT Harmonization Taskforce” (HarT) comprised implementers (n = 8), manufacturers (n = 4) and regulatory experts (n = 19); its mandate included finalization of the suggestions for interchangeability and user-friendliness of RDTs. Between February and June 2014, HarT refined the priorities for labelling and IFU and delivered consensus documents including a list of harmonized terminology and abbreviations, specifications for labelling of RDT box, buffer bottle, device packaging and accessories, as well as a generic template for IFU.

### Ethical clearance

The project was submitted to and approved by the Institutional Review Board of the Institute of Tropical Medicine (851/12).

## Results

### Working document of best practice suggestions for labelling including IFU of malaria RDTs

**Figure 1:**
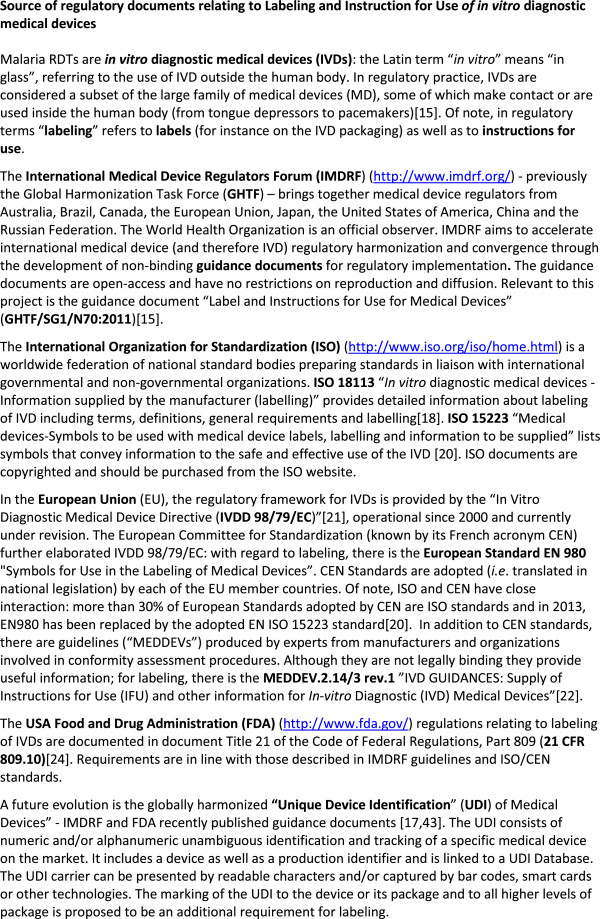
**Source of regulatory documents relating to labelling and instruction for use** ***of in vitro*** **diagnostic medical devices**.

Figure 1 gives an overview of regulatory documents relating to labelling and IFU for IVDs. Standards and norms, guidelines and suggestions were assessed according to the following subjects: general requirements, content of the label, specific labelling of RDT box, packaging, buffer bottle, cassette and accessories, and IFU. Based on their assessment, the final working document included 66 suggestions of which more than two-thirds (n = 46, 70%) were related to labelling and IFU [26]. Of these 46 suggestions, 33 (71.7%) were sourced from regulatory documents and/or published literature, another 12 were made by implementers and the remaining one was made by a manufacturer. In interviews with manufacturers, most suggested requirements (33/46, 72%) were scored as “highly relevant” and “feasible to align with in the short term” (*i.e.* within one year).

### Outcomes of consultation with RDT implementers, manufacturers and experts in regulatory matters

The recommendations compiled in the working document were modified and refined during the consultation meeting. Some implementers’ suggestions were not seen as critical, amongst them the recommendations (i) to use an identical lot number for box, cassette packaging and buffer vial, (ii) to display a version number of IFU on the RDT box or device packaging, (iii) to add a revision history to the IFU and (iv) to affix the date of production (except in case of national regulatory requirement). Likewise, the recommendations to display all essential information (including custom/variable information) grouped together on at least two sides of the box and to print all text and symbols in the same direction (to facilitate visual capture at inspection) were withdrawn as well as a minimum interline space of 2 for print formatting for the IFU. The consultation report was published on the RBM website [28].

Interviews with implementers confirmed that the plethora of RDT products was perceived as a major operational problem, because of variations in test procedures and because of differences in reading and interpretation of results (*e.g.* the characters used for the reading legend and for the labelling of the sample and buffer wells).

### mRDT Harmonization task force (HarT) consensus documents

**Figure 2:**
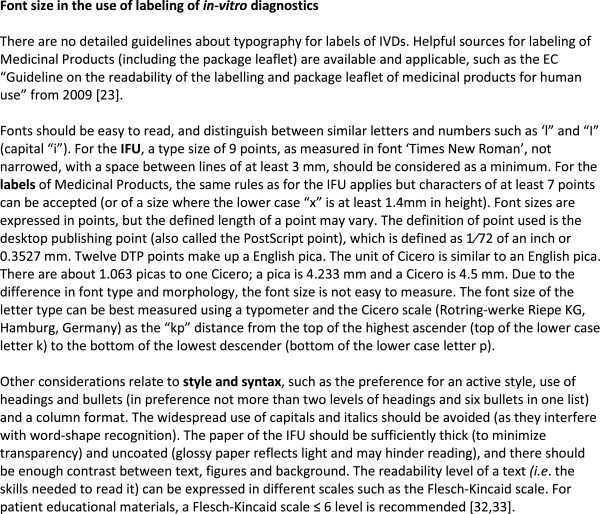
**Font size in the use of labelling of** ***in-vitro*** **diagnostics.**

**Figure 3:**
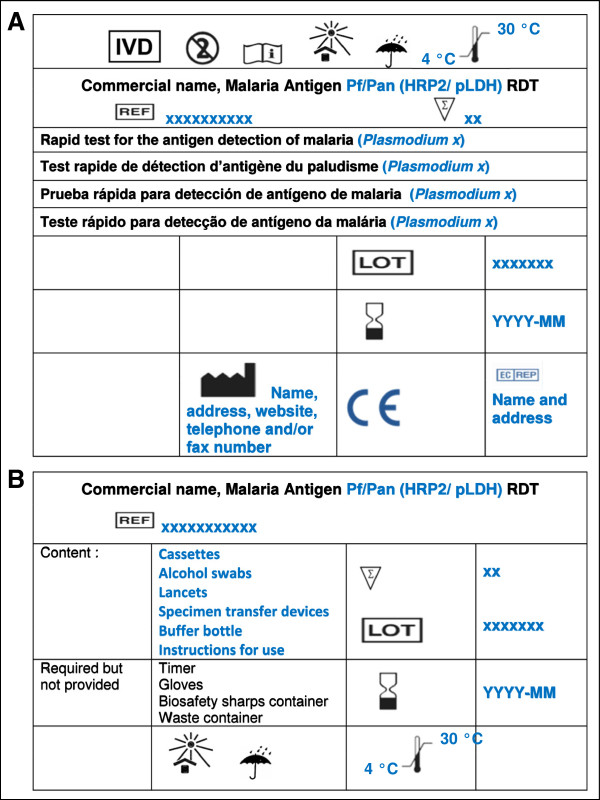
**Examples of the required labelling of the RDT box.** 3 **A**: top side, 3 **B**: lateral side. Labelling includes product name, product code, intended use, number of test, information about the manufacturer, symbols addressing storage conditions, warnings and precautions. Text in blue displays RDT product specific characteristics.

The HarT further elaborated the consultation report and developed consensus documents for RBM, presented as additional files herein. Additional file 1 lists the recommended terms and abbreviations related to malaria RDTs, Additional file 2 lists the recommended requirements for labelling of box, cassette packaging, cassettes, buffer bottle and accessories. Emphasis was placed on durability (permanent printing or water-resistant labels lasting the life-span of the RDT product), legibility (font size, letter type, see Figure 2), comprehension (use of symbols) and ease of reference (*e.g.* place of labelling on the box or cassette packaging, allowing quick oversight). HarT further recommended the use of the official language(s) of the country/region of intended use (expert suggestion). In addition, it was proposed to foresee a place for affixing the Unique Device Identification (UDI, see Figure 1) label (expert suggestion). Among the information required to be displayed, a meaningful product name (revealing the intended use and antigens targeted) was recommended and (as for other RDT components), the expression of the expiry date was agreed as year and month (YYYY-MM). The information identifying the legal manufacturer should include the name, the physical address of the manufacturing site and contact details, *i.e.* telephone and/or fax number and website. HarT recommended also to display a clearly visible additional warning on the RDT box in case the test procedure or IFU had changed substantially. Figure 3A and B display examples of the required labelling of the RDT box, including product name, product code, intended use, number of tests, information about the manufacturer as well as symbols addressing storage conditions, warnings and precautions. All relevant information needed for stock management (*e.g.* product identity, storage conditions and materials provided) should also be displayed on two sides, *i.e.* the front side and at least one lateral side of the RDT box, except for custom/variable information such as lot number and expiry date which could be (for reasons of cost) printed on only a single side of the box (implementer suggestion).

Likewise, consensus on labelling specifications for buffer bottle, cassette packaging, desiccant packaging and accessories (specimen transfer devices, lancets, alcohol swabs and desiccants) were compiled (Additional file 2, Figure 4). Similar recommendations for labelling relating to durability, legibility and user-friendliness were made. For the cassette packaging, the consensus was to display all standard information (name, storage, warnings and precautions) on one side of the packaging and to display the custom or variable information (lot number, expiry date) on the other side; this to allow cost-effective printing and processing (implementer suggestion).

**Figure 4:**
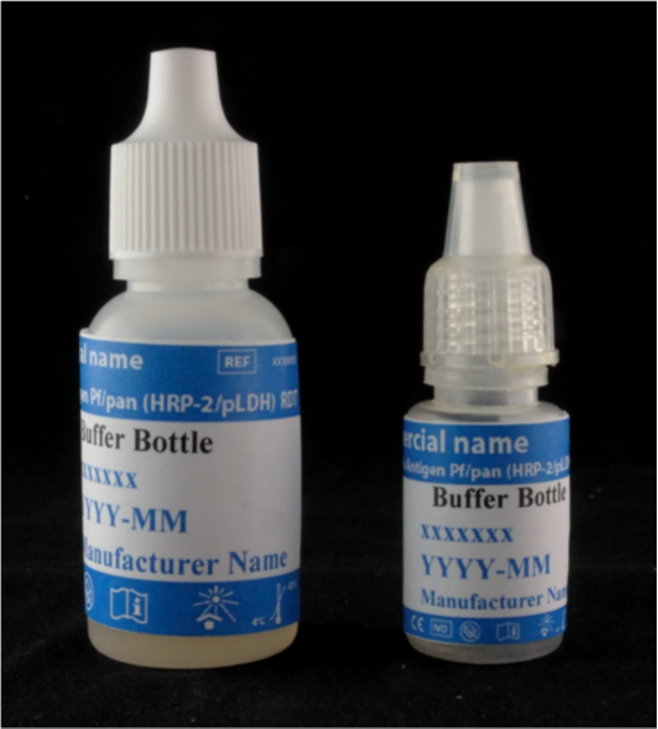
**Labelling of the buffer bottles.** Two common sizes of buffer bottles, labeled according to the HarT requirements. The label used for the large buffer bottle has a size of 4.9 cm × 3.0 cm; all information is displayed in a font Times New Roman, size 10 points, bold. For the small buffer bottle, a label of 4.9 cm × 2.3 cm is used. Essential information (commercial name, buffer bottle, expiration, lot number) is printed in Times New Roman bold, size 9 points; other information is printed in Times New Roman bold size 7 points.

For the accessories, HarT referred to the option to display the required information on the packaging of multiple devices when it is not practicable to display it on the device itself [15, 21]. Lancets and alcohol swabs are regulated as medical devices and are to be labeled as such; alcohol swabs are to be labeled as antiseptics (contact with tissues), not as disinfectants (contact with objects).

**Figure 5:**
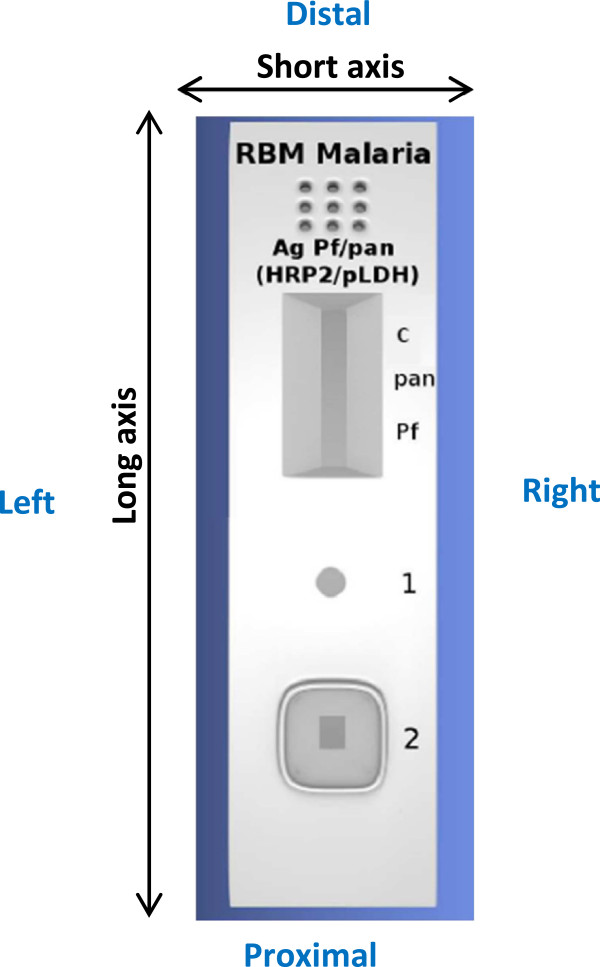
**Conventions for terms of the cassette.** This figure considers the most common malaria RDT *i.e.* a three-band RDT targeting two antigens (*P. falciparum* and pan-*Plasmodium* antigen) in a two-step procedure (add specimen, next add buffer) with a cassette showing separated specimen and buffer wells. The following convention of terms is used: proximal (closest to the specimen and buffer wells) and distal (at the end of the migration [absorption] pad). Considering the cassette in a vertical view (with direction of the specimen/buffer flow “upwards”), there is the right hand side and the left hand side.

**Figure 6:**
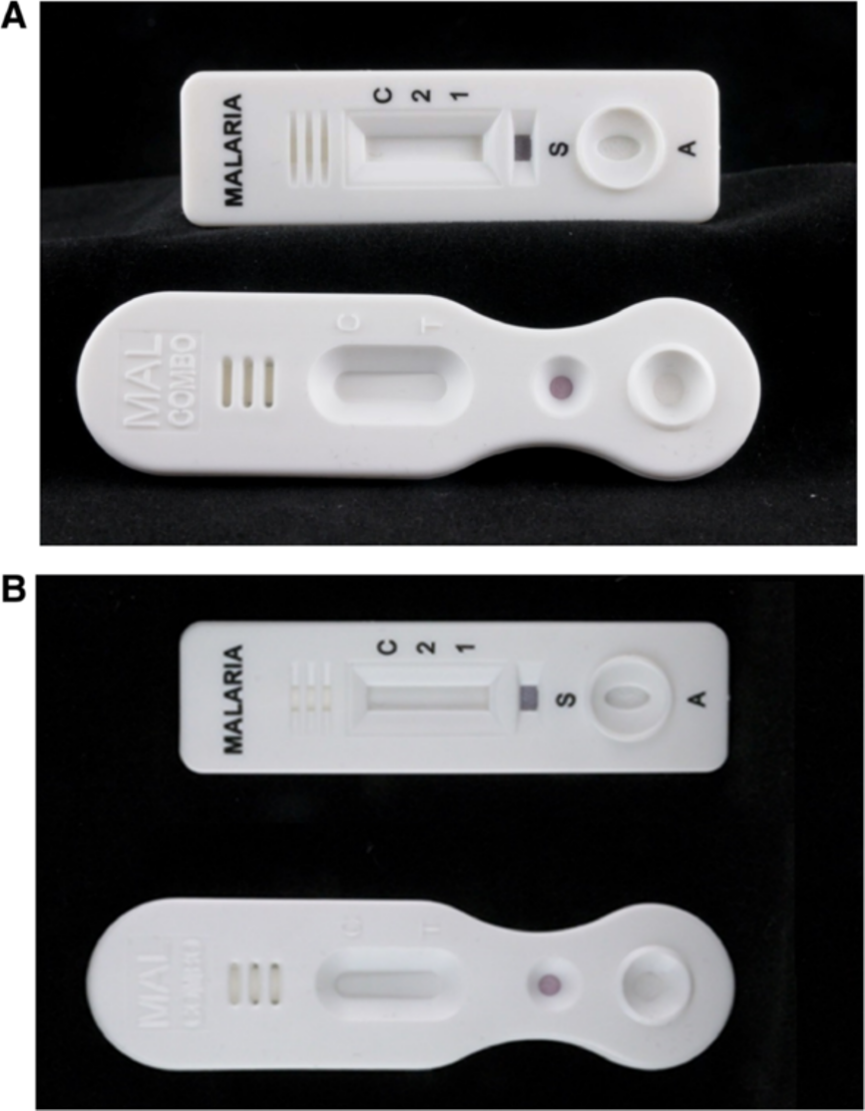
**Malaria RDT cassettes: differences in visibility between embossed versus printed characters.** The figures demonstrate the difference between visibility/readibility of embossed versus printed characters. **A** shows the cassettes in tangential (“side”) light: the embossed characters are well discernable. **B** shows the cassettes in regular (direct) light: the embossed characters are less discernable – visibility will even decrease in low light conditions.

For labelling of the cassette, a convention for terminology and orientation of labelling was agreed (Figure 5, implementer suggestion). Prints in indelible ink are recommended over characters embossed in the cassette housing or labels glued to the reading legend (Figure 6). Orientation of the text should be parallel to the short axis, and a single unequivocal reading legend should be displayed at the right hand side of the results window. The product name should be printed on the distal side of the cassette. Specimen and buffer wells should be labeled as “1” and “2” respectively, according to the chronological order of the procedure. *Plasmodium* species detected should be displayed by the agreed abbreviation in the reading legend (Additional file 1).

A final HarT consensus document addressed the specifications for the IFU in the form of a generic template with background information and detailed instructions (Additional file 3). The instructions addressed the lay-out and style and its user-friendliness (including referral to tools for assessing readability level) as well as a referral to relevant and updated websites and studies. Recommendations were also compiled about appropriate description of IVD performance specifications. In addition an example was provided of a symbol key displaying the internationally recognized symbols relevant for IVDs, along with their explanation (expert suggestion).

## Discussion

### Labelling assists performance, quality and safety of in vitro diagnostic medical devices (IVDs)

Regulation of IVDs targets consistent performance and quality of products and their safety. Best international regulatory practice dictates that a manufacturer should undertake an ongoing risk assessment of the IVD product’s use and manufacture, in which all risks and their consequences (false or missed diagnosis, transmission of infections etc.) are identified and minimized by appropriate design, construction and manufacturing [17, 21]. Communication of any residual risk to the user has to be provided by labelling, which includes labels as well as the IFU (Figure 1) [15, 17]. As such, this communication includes warnings and precautions but also statements of known test limitations.

### Labelling should target the user’s profile

An explicit part of the regulatory responsibility of the manufacturer is to align communication to the level of the IVD user’s education, training and expertise [15, 17]. Medium, format, content, legibility and location of the label should be appropriate for the intended user [15]. This has particular relevance when considering the use of malaria RDTs in the private sector, the community and home-based management of malaria [4, 29–31].

### Guidelines for lay-out and readability, and the use of internationally recognized symbols

There are no guidelines defining labelling font sizes and styles for IVDs, but useful information can be found in pharmaceutical guidance on labelling of medicines’ package leaflets referring to font, lay-out and style (Figure 2) [23]. In terms of syntax, readability is expressed by so-called reading scales such as the Flesch-Kincaid grade which expresses the U.S. grade-level equivalence of the skills required to read a particular document: for the purpose of RDTs in the present setting, a grade ≤ 6 is recommended, in line with the requirements for patient educational materials [32, 33].

In view of swift and consistent interpretation of communication, the use of graphical symbols is promoted: symbols save space, obviate the need for translations and convey standardized and clear messages at high visual impact and noticeability [15, 20, 21, 34]. Symbols described in ISO 15223 [19] may be used in CE marked IVDs without further explanation, however, comprehension of these assumed “self-explanatory” symbols may be poor particularly among untrained staff in resource limited settings [35]. Therefore, in addition to training and educational outreach, adding a symbol key (*i.e.* a glossary of symbols used) to the IFU is recommended, in line with FDA recommendations [36].

### Relevance for the RDT market

The malaria RDT market currently comprises a plethora of products and manufacturers, with constant revisions made to products in order to improve quality and performance [13, 14]. Although the public sector is centered around a core set of products and suppliers, this changes over time. Products in the private sector are more diverse and mostly not aligned with those products recommended by WHO and in turn, the NMCPs (Availability and price of malaria rapid diagnostic tests in the private health sector in 2011: results from ten nationally representative cross-sectional retail surveys. Poyer S., Goodman C et al., personal communication, [5]). Lack of quality has been noted as an important market shortcoming: the RDT market has started in an era with minimal regulatory oversight or quality standards and most resource limited settings have no effective IVD regulations or post-market surveillance in place [5]. Despite quality initiatives such as the WHO Malaria RDT Product Testing [37] and WHO-FIND Lot Testing Programmes [38] the WHO RDT Procurement Guidance [14] and the WHO Prequalification of Diagnostics and Medical Devices Programme [39], the RDT market is mainly driven by the economics of scale [5]. Prices and benefit margins have declined over recent years and high orders, short lead times and an unpredictable market may compromise product quality [5].

Another perceived difficulty of RDT use was confirmed by interviews with implementers from the public sector in the present study but also recorded from the private sector [40]: weaknesses referred to aspects of labelling and IFU, as well as RDT accessories (lancets, alcohol swabs and specimen transfer devices). Differences in these aspects contribute to the difficulties encountered in changing from one RDT product to another, a practice dictated by the open competitive tenders in the RDT market [5].

The present study assessed the available evidence to identify best practices in labelling and IFUs tailored to malaria RDTs and associated accessories. In the initial phases of the project, identifying a representative group of implementers involved in daily use of RDTs was challenging; however, the consultation on the original working document [26] and subsequent iterative review processes gradually included a large number of participants whose activities and roles are highly related to malaria RDTs use in the field, as well as their manufacturing, their procurement and use, and their regulation. Should this set of recommendations, most of which are derived from existing international guidelines, be adopted broadly, it is expected to make RDTs more user friendly and to facilitate product interchangeability. Moreover they may contribute to increased quality and improved performance of RDTs. Based on the information of participating manufacturers, most – if not all – recommendations can be achieved at a reasonable cost and in a short time span. In addition to producing a meaningful list of recommendations and reference documents, this study has also stimulated the dialogue between users, implementers, buyers, regulatory experts and manufacturers. The consensus document generated through this collective process steered by RBM [28], may also be of value to rapid diagnostic tests and IVDs addressing other infectious diseases which is relevant since most manufacturers have RDTs in their portfolio specific for multiple diseases.

### The way forward, benefits of harmonization/labelling

The present HarT recommendations offer an opportunity for improving consistency and harmonization of RDT product characteristics and provide a comprehensive resource upon which international recommendations and tender specifications can be based. Various channels may be exploited for diffusion and application to ensure maximum uptake. Furthermore, the recommendations may also guide and orient emerging national IVD regulations and regional initiatives such as the Pan-African Harmonization Working Party on Medical Devices and Diagnostics (PAHWP) [41]. Synergistic integration of the recommendations into assessment procedures and procurement practices – particularly when extended to other IVDs - will add quality as a driving market factor, increase awareness about quality standards for IVDs among end-users and provide incentives to manufacturers to further invest in robust quality systems.

## Conclusions

The present document together with its additional files compiled proposes best practices in labelling and IFU for malaria RDTs. It is expected that compliance with these best practices will increase harmonization among the different malaria RDT products available on the market and improve their user-friendliness.

## Electronic supplementary material

Additional file 1: **Suggested terms and abbreviations related to malaria RDTs.** (PDF 74 KB)

Additional file 2: **Requirements for the labelling of malaria RDT kit components: box, cassette packaging, cassette, buffer bottle and accessories.** (PDF 719 KB)

Additional file 3: **Generic template for Instructions for Use (IFU).** (PDF 402 KB)

## Abbreviations

CLSI: Clinical and Laboratory Standards Institute
EC: the European Commission
FDA: Food and Drug Administration
GHTF: Global Health Task Force
HarT: Harmonization Task force
IFU: instructions for use
IMDRF: International Medical Device Regulators Forum
ISO: International Organization of Standardization
ITM: Institute of Tropical Medicine
IVD: *in vitro* diagnostics
mRDT: Malaria rapid diagnostic test
NMCP: National Malaria Control Programme
P: *Plasmodium*
PAHWP: Pan-African Harmonization Working Party on Medical Devices and Diagnostics
RBM: Roll Back Malaria Partnership
RDT: Rapid diagnostic test
UDI: Unique device identification
WHO: World Health Organization.
