# Supplementary material for: Harmonization of malaria rapid diagnostic tests: best practices in labelling including instructions for use
Source: Malar J. 2014 Dec 17;13:505. doi: 10.1186/1475-2875-13-505 (PMC4302121; doi:10.1186/1475-2875-13-505)
Supplement: Supplementary file 1 — Additional file 1: Suggested terms and abbreviations related to malaria RDTs. (PDF 74 KB) [file 12936_2014_3663_MOESM1_ESM.pdf]

Additional file 1. Suggested terms and abbreviations related to malaria RDTs

1. Recommended terms

Preferred Term	Description	Comments/synonyms (not suggested term)
Accessories	Articles intended explicitly by its manufacturer to be used together with the RDT to achieve its intended purpose (*i.e.* specimen transfer device, lancet, alcohol swab).	Synonym: “ancillary items”
Alcohol swab	Gauze pad that is saturated with alcohol and used to clean and/or disinfect skin.	Synonym : "alcohol pad, alcohol wipe, alcohol pre pad"
Auto-transfer cassette	Cassette presenting with an opening which allows direct sampling of the blood on the nitrocellulose strip.	
Buffer	Buffered protein and detergent solution to enable specimen flow, to optimize sensitivity and minimize non-specific reactions.	Many synonyms are in use (*e.g.* “blood lysis buffer “, “clearing buffer”, "assay diluent", "sample diluent", "reagent" etc.)
Buffer bottle	Plastic bottle with cap and nozzle, containing the buffer, intended to be used for the RDT tests packed in one RDT kit.	
Buffer vial	Small vial containing the volume of buffer needed to perform a single RDT test. Mostly used in RDTs packed as single pack (and/or marketed for self-testing).	Synonym: "buffer ampulla"
Buffer well	Physical place in the test device, where the buffer is applied.	
Cassette	Test device with the nitrocellulose strip encased in a plastic housing, and presenting openings for the result window, for the specimen and buffer wells and in some cases to allow evaporation.	
Combination rapid diagnostic test	Malaria rapid diagnostic test detecting more than one antigen (different combinations possible).	
Component	Part of a finished, packaged and labeled RDT kit. The necessary components to perform the RDT test include: test device, buffer bottle, specimen transfer device, lancet, alcohol swab and instructions for use.	
Control line	Visible line on the nitrocellulose strip that indicates that the migration was satisfactory.	
Desiccant	Drying agent used to protect the test device from humidity.<br>The desiccant can be self-indicating (all beads coated with a humidity indicator that changes color upon saturation), partial-indicating (part of the beads colored) or non-indicating (no colored beads).	Silica gel is the most commonly used desiccant for RDT products.

Preferred Term	Description	Comments/synonyms (not suggested term)
Device	Physical platform of the RDT such as cassette, hybrid, dipstick.	Use this term when referring to any type of test device, when speaking about/referring to a particular device (such as "cassette"), use this term and not "device"
Dipstick	Test device consisting of only the nitrocellulose strip that is be placed on/in a platform (tube, backing paper…).	The term "dipstick" actually refers to non-migrating tests such as test for detection of glucose in urine, but is commonly used to indicate the malaria lateral flow test as explained here.
End-user	The person, trained or skilled, who uses a medical device.	
Hybrid	Test device consisting of a nitrocellulose strip to be dipped into a plastic housing with buffer and specimen wells.	
in vitro diagnostic (IVD) medical device	A device, whether used alone or in combination, intended by the manufacturer for the *in vitro* examination of specimens derived from the human body solely or principally to provide information for diagnostic, monitoring or compatibility purposes. This includes reagents, calibrators, control materials, specimen receptacles, software, and related instruments or apparatus or other articles (International Medical Devices Regulators forum).	http://www.imdrf.org/docs/ghtf/archived/sg1/technical-docs/ghtf-sg1-n045r12-in-vitro-diagnostic-classification-070209.pdf
Instructions for use (IFU)	Information provided by the manufacturer to inform the end-user of the IVD's intended purpose and proper use and of any precautions to be taken. (GHTF/SG1/n70:2011)	Synonym: "package insert"
Job Aid(s)	Document describing the essentials to perform an RDT test (*i.e.* Procedure/Interpretation) and provided apart from the IFU, either as a separate leaflet, and/or printed on the device packaging or in/on the RDT box.	Refer to the "WHO generic job aids" as an example.
Kit	Set of components that are co-packed together and intended to be used to perform a specific RDT (Test device, buffer bottle, specimen transfer device, lancet, alcohol swab, instructions for use) (adapted definition from EN ISO 18113-1:2009)	

Preferred Term	Description	Comments/synonyms (not suggested term)
Lancet	Sharp needle-like device used to puncture skin to obtain blood (CLSI H04-A6)<br>This includes:<br>- Plain metal lancet (for reasons of sterility packed into a single packaging)<br>- Safety-seal lancets (lancets in plastic housing, with a plastic cap)<br>- Auto-lancing lancets (lancet mounted in plastic housing that is automatically ejected when plunger is pressed)<br>- Auto-retractable lancets (lancet mounted in plastic housing that is automatically retracted after puncture)	
Lot	Defined amount of material that is uniform in its properties and has been produced in one process or series of processes, so that it could be expected to be homogeneous. (The material can be either starting material, intermediate material or finished product) (EN ISO 18113-1:2009)	Synonym: "batch"
Lot number	Distinctive set of numbers and/or letters that specifically identifies a lot and permits its manufacturing, packaging, labeling and distribution history to be traced (EN ISO 18113-1:2009)	Synonyms: "batch number", "batch code"
Malaria RDT	A collection of reagents and other associated materials intended to be used for the qualitative and/or quantitative detection of antigens from one or multiple species of Plasmodium (malaria parasites) in a clinical specimen within a short period, relative to standard laboratory testing procedures, using an immunochromatographic test (ICT) method. This test is commonly used in the laboratory or in point-of-care analyses.	
Manufacturer	Any natural or legal person with responsibility for design and/or manufacture of a medical device with the intention of making the medical device available for use, under his name; whether or not such a medical device is designed and/or manufactured by that person himself or on his behalf by another person(s). (GHTF/SG1/N055: 2009 Definitions of the Terms Manufacturer, Authorized Representative, Distributor and Importer).	
Plasmodium antigens	Antigens produced by malaria parasites and detected with RDTs.	
Point of care testing (POCT)	Testing used at or near the site of patient care, with the result leading to possible change in the care of the patient.	

Preferred Term	Description	Comments/synonyms (not suggested term)
(Primary) Packaging of alcohol swab, alcohol swab packaging<br>(Primary) Packaging of lancet, lancet packaging<br>(Primary) Packaging of desiccant, desiccant packaging<br>(Primary) Packaging of cassette, cassette packaging	Primary packaging of the alcohol swab, the plain metal lancet and the desiccant.<br>Use "Primary packaging" when addressing technical comments and manufacturers (for instance when describing requirements for labeling) and use simply "Packaging of the alcohol swab/alcohol swab packaging", "Packaging of the lancet/lancet packaging", "Packaging of the desiccant/desiccant packaging" , "Packaging of the cassette/cassette packaging" etc. when addressing the end-user in trainings and IFU (for instance: open the "cassette packaging ") .	Synonym: "pouch", "sachet"
Product	RDT as currently marketed and identified, with assigned name, product code/regulatory version.	
Product code	Code identifying a product with assigned name and regulatory version.	Synonyms : "catalogue number", "product number", "reference number"
Rapid diagnostic test (RDT)	Single use IVD designed for use at the point-of-care (POC) and fulfilling the ASSURED criteria (affordable, sensitive, specific, user-friendly, rapid and robust, equipment-free, delivered to those who need it).	
RDT box or "box"	Physical box, mostly produced out of cardboard, in which the kit components are packed.	Synonym: "secondary packaging"
Reading legend	Scale of acronyms or characters along the result window, referring to the control and test lines. The characters may be embedded in the plastic housing or printed/glued on it. The reading legend can be at either side of the result window, sometimes two reading legends (each at one side of the result window) are displayed.	Synonym: "reading scale"
(Minimum and maximum) reading time	Time at which result readings must be done	
Result window	Opening in the test device showing the area of the strip containing the control and test line(s).	Synonym: "reading window"
Revision history	A table to record the amendments made each time a new version of the IFU is issued.	
Self-test	Any device intended and stated by the manufacture to be used by lay persons in a home environment for self-diagnosis purposes. (98/97/EC)	
Single pack	RDT individually packed with all components needed for the performance of one test.	Synonyms: single test pack, single test, individually packaged test

Preferred Term	Description	Comments/synonyms (not suggested term)
Specimen well	Physical place in the test device, where the specimen is applied.	
Specimen transfer device	Device used to transfer blood to the test device.<br>This includes: inverted cup, loop, (glass) capillary tube, (plastic) straw, pipette.	The term "specimen transfer device" may be shortened to "transfer device".<br>Synonym: "sampling device"
Symbol key	List of symbols with written explanation ("legend").	Synonym: "key to symbols"
Temperature indicator/monitor	Simple, inexpensive smart label that shows the temperature or the time/temperature history of the product to which it is attached to during shipping or storage.	Synonym: "temperature tag"
Test device	Physical component that contains the platform where the reaction takes place.	Test device formats: cassette, dipstick, hybrid, auto-transfer cassette.
Test line	Visible line on the nitrocellulose strip displaying the reaction of a specific target antigen (HRP2, pLDH, aldolase, etc.).	The term "band" can be used when referring to the RDT design (two-, three- and four-band RDT products)
Test strip	Nitrocellulose strip which is the platform of the lateral flow immunochromatographic test.	Synonym: "test membrane"
Two-, three- and four-band RDTs	RDTs consisting of two, three or four lines, consisting of the migration control line and one, two or three test lines respectively.	
Unique Device Identification(UDI)	The UDI is a series of numeric or alphanumeric characters that is created through a globally accepted device identification and coding standard, which allows the unambiguous identification of a specific medical device on the market. The UDI comprises the Device Identifier and Production Identifier (including serial number, lot number, manufacturing and/or expiration date).	
Version number	Number given to the instructions for use (or job aids) to allow to track changes.	

2. Abbreviations

Abbreviations	Meaning	Comments
C	Control	
HRP2	(*P. falciparum*) Histidine-Rich Protein2	
IFU	Instructions for use	
P.	*Plasmodium*	“P” is only to be used as abbreviation of the genus and in combination with the species (e.g. *P. ovale*) or as part of the abbreviations “Pm, Po, Pv, Pf and Pvom), so not as stand-alone.
pan-	a group of *Plasmodium* species: Pf, Pv, Po and Pm	
Pf	*Plasmodium falciparum*	
pLDH	*Plasmodium* (or parasite) Lactate Dehydrogenase	In papers and documents, both parasite-Lactate Dehydrogenase and *Plasmodium* Lactate Dehydrogenase are used - in the present scope and documents (such as IFU) it makes more sense to the user to maintain “*Plasmodium*”
Pm	*Plasmodium malariae*	
Po	*Plasmodium ovale*	
Pv	*Plasmodium vivax*	
spp.	Species	
Pvom	*P. vivax, P. ovale, P. malariae*	
