# Supplementary material for: Harmonization of malaria rapid diagnostic tests: best practices in labelling including instructions for use
Source: Malar J. 2014 Dec 17;13:505. doi: 10.1186/1475-2875-13-505 (PMC4302121; doi:10.1186/1475-2875-13-505)
Supplement: Supplementary file 2 — Additional file 2: Requirements for the labelling of malaria RDT kit components: box, cassette packaging, cassette, buffer bottle and accessories. (PDF 719 KB) [file 12936_2014_3663_MOESM2_ESM.pdf]

Additional file 2. Requirements for the labeling of malaria RDT kit components: box, cassette packaging, cassette, buffer bottle and accessories

1. Labeling of the RDT Box

Orientation:

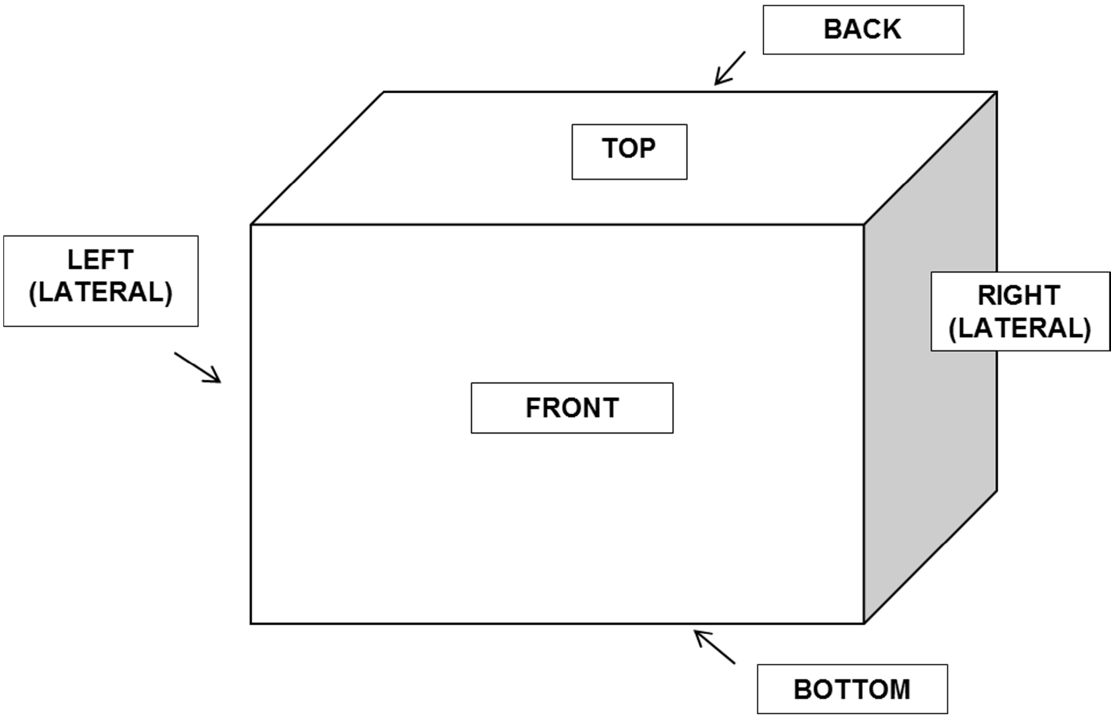

Figure 1: Convention of terms for the front-view of a malaria RDT box.

General requirements and outlines for labeling of malaria RDT boxes are as follows:

1. **Labels:** Labels should be printed on the cardboard as permanent printing or applied as water-resistant labels (applied with water resistant glue). Prints should be indelible and should last the life span of the RDT product.

2. **Use of Symbols** is encouraged. Only internationally recognized symbols (EN ISO 15223 – 2012) should be used.

3. **Labeling must be legible**: for instance open letter type and font size equivalent to Miriad bold 10.

4. The official **language(s)** in which the intended use is displayed, should be relevant to the region where the RDT product will be used. In the example below, English, French, Spanish and Portuguese are displayed.

5. Foresee a place for affixing the carrier (label) of the so-called **UDI (Unique Device Identification)**. The UDI is a series of numeric or alphanumeric characters created through a globally accepted device identification and coding standard, which allows the unambiguous identification of a specific medical device on the market. The UDI comprises the Device Identifier and Production Identifier (including serial number, lot number, manufacturing and/or expiration date). On the Bluebox model, place is foreseen for the UDI.

6. Display the essential information (see below) on the **front side and at least one lateral side of the RDT box (left or right).** The information contains all relevant information needed for stock management (*e.g.* product identity, storage conditions and material provided). An exception can be made for custom/variable prints such as lot number and expiration date and in case of use, also production date – they can be printed on only a single side of the box.

What should be displayed:

1. Product name with sufficient details for the user to identify the device and its intended use, *e.g.*

- Commercial name of the RDT product
- "Malaria"
- targeted species and antigen(s)
- "Antigen" or "Ag"

Example: Commercial name, Malaria Pf/Pv (HRP2/pLDH) Antigen(RDT)

2**. Product code**

3. **Intended use** : diagnosis of malaria, *in vitro* diagnostic, single-use

4. **Number of tests** provided in the kit

5. ***In vitro* diagnostic**

6. Name of the **legal manufacturer**
Physical address of the manufacturing site
Telephone and/or fax number and/or website

7. If applicable (for instance CE-marked IVDs), the name and physical address of the authorized representative. Affixing name and address of local distributor/importers is desirable, to be done by the distributor/importers themselves.

8. **Lot number**

9. **Expiration date** (YYYY-MM)
Use the YYYY-MM format

10. Materials (content)
Materials provided
Items required but not provided

11. **Storage conditions** (symbols)

12. **Warnings or precautions** (symbols)
For instance: - do not use if package is damaged (symbol)
- read instructions before use (symbol)

13. **Additional warning in case the procedure or IFU has changed substantially**
Clearly visible label – for instance fluo orange

Where should labels be put:

Display the essential information on at least the front side and one lateral side of the box. An exception can be made for custom/variable prints such as lot number and expiration date and in case of use, also production date – they can be printed on only a single side of the box. See Figures 2 and 3. CE label, EC authorized representative and date of manufacturing should be printed when applicable/required.

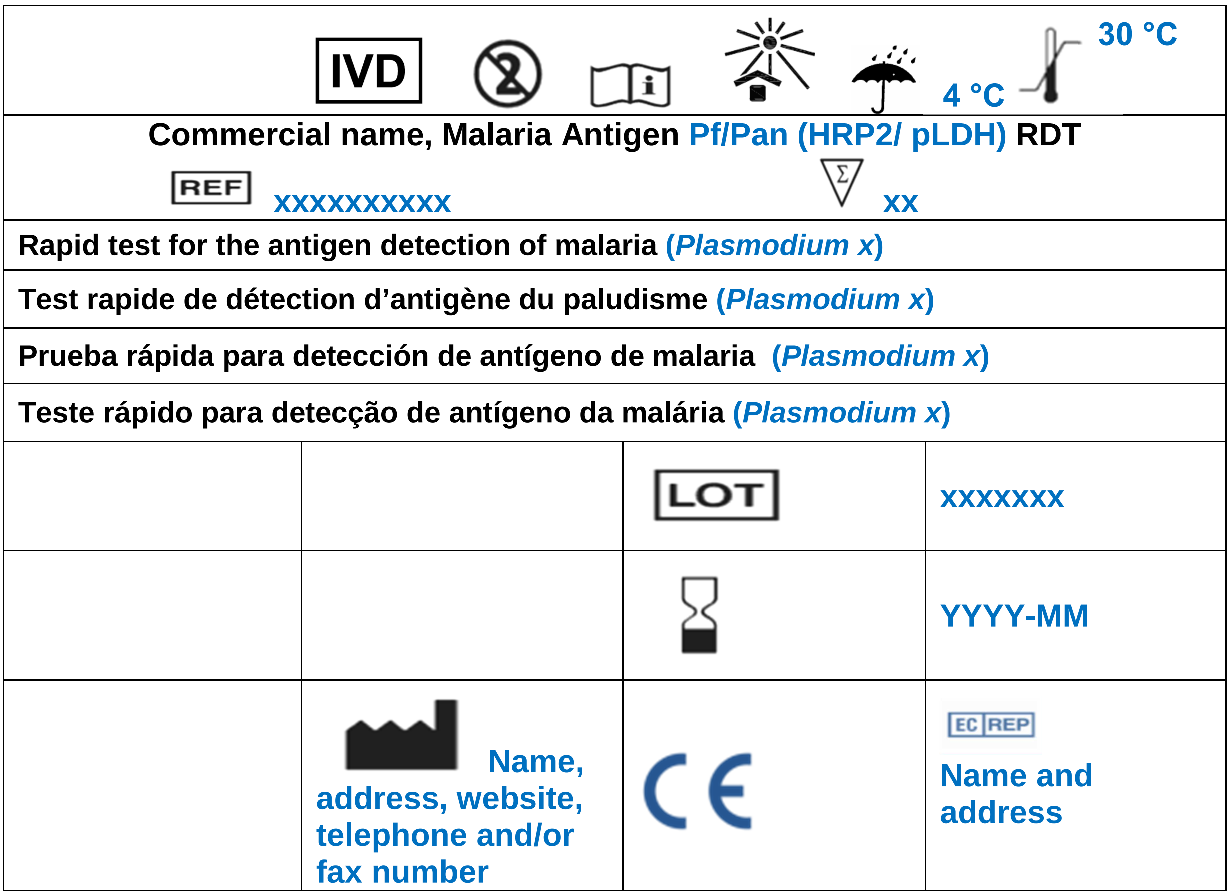

Figure 2: Labeling of required information on the top of the RDT box. Blue color indicates RDT product specific items.

Commercial name, Malaria Antigen Pf/Pan (HRP2/ pLDH) RDT			
REF xxxxxxxxxxx			
Content :	Cassettes<br>Alcohol swabs<br>Lancets<br>Specimen transfer devices<br>Buffer bottle<br>Instructions for use	 LOT	xx<br>xxxxxxx
Required but not provided	Timer<br>Gloves<br>Biosafety sharps container<br>Waste container		YYYY-MM
		4 °C 30 °C	

Figure 3: Proposal for labeling of one lateral side, and the front or back side of the RDT box. Blue color indicates RDT product specific items.

2. Labeling of the cassette packaging

What should be displayed:

1. Product name with sufficient details for the user to identify the product and its intended use, *e.g.*

- Commercial name of the RDT product
- “Malaria”
- targeted species and antigen(s)
- “Antigen” or “Ag”

Example: Commercial name, Malaria Pf/Pv (HRP2/pLDH) Antigen(RDT)

2. **Product Code**

3. **Intended use**: diagnosis of malaria, *in vitro* diagnostic, professional use only, POC

4. ***In vitro* diagnostic**

5. Name of the legal manufacturer

6. **Lot number**
The lot number should be identical to the one mentioned on the RDT box

7. **Expiration date** (YYYY-MM)
Use the YYYY-MM format
The expiration date should be identical to the one mentioned on the RDT box

8. **Quantity of tests per packaging**

9. Contents of packaging
Including desiccant

10. **Storage conditions** (symbols)

10. **Warnings or precautions** (symbols)
For instance: - do not use if package is damaged (symbol)
- read instructions before use (symbol)
- single use (symbol)

11. If the RDT product is CE-marked, the device packaging should have (and comply with) the **CE mark** too

Where should labels be put:
Display all standard generic information on one side of the packaging and the custom/variable information (expiration date, lot number) on the opposite side.
Blue color in the figure below indicates RDT product specific items.

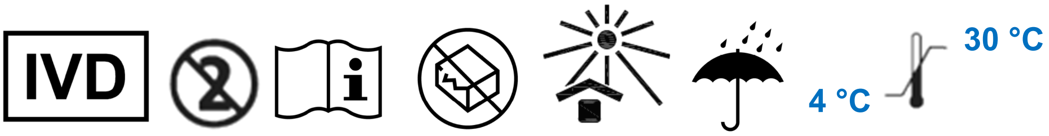

**Commercial name, Malaria Antigen Pf/Pan (HRP2/ pLDH) RDT**	
**Product code: XXXXXXX**	
Σ	**xx**
Content:	- 1 cassette<br>- 1 desiccant<br>- 1 specimen transfer device
	**Manufacturer Name**
CE	

LOT	**xxxxxxx**
	**YYYY-MM**

3. **Labeling of the cassette**

Convention of terms used to describe the orientation of the cassette

The figure below considers the most common RDT *i.e.* a three-band RDT targeting two antigens (*P. falciparum* and pan-*Plasmodium* antigen) in a two-step procedure (add specimen, next add buffer) with a cassette showing individual specimen and buffer wells. The following convention of terms is used: proximal (closest to the specimen and buffer wells) and distal (at the end of the migration [absorption] pad). Considering the cassette in a vertical view (with direction of the specimen/buffer flow "upwards", there is the right hand side and the left hand side.

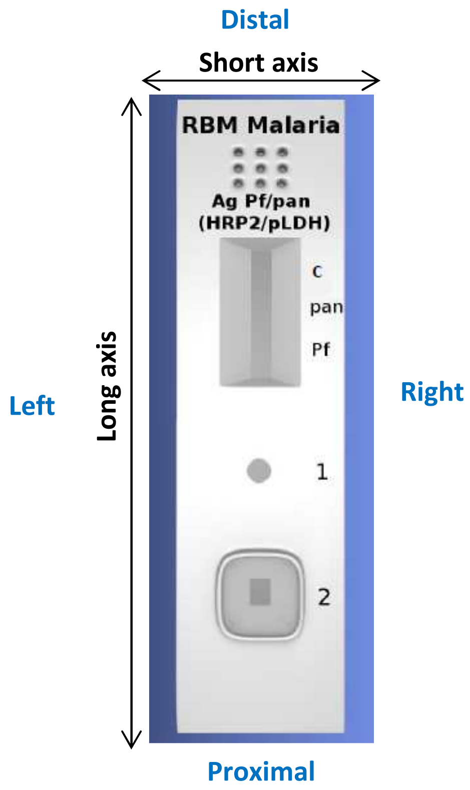

Figure: Conventions for terms of the cassette.

Labeling:

1. Prints in **indelible ink** are recommended over (i) characters embedded/embossed in the cassette housing and (2) labels glued to the reading legend. The test and control line legends and the actual test lines should be well aligned.
2. All printing should be along the **short axis**
3. **Single and unequivocal reading legend** should be present **at the right hand side** of the reading window
4. Abbreviations as listed in document "**Abbreviations**"
   In addition "1" for the sample well, "2" for the buffer well (chronological order)
5. **Labeling must be legible**: for instance open letter type, clear and crisp print

What should be displayed:

1. Product Name (with indication of "Malaria", antigen-based "Ag", the *Plasmodium* species and the antigens detected)
2. Specimen and Buffer wells (see above)
3. Reading legend with *Plasmodium* species detected (see abbreviations: Pf, pan, Pv…)

4. **Labeling of the buffer bottle**

General requirements and outlines for labeling of RDT **buffer bottle** is as follows:

1. **Labels:** Well-fixed water-resistant label (applied with water resistant glue) or permanent printing, indelible print lasting the life span of the RDT product.

2. **Use of Symbols** is encouraged. Only Internationally recognized symbols (EN ISO 15223 – 2012) should be used.

3. **Labeling must be legible**:
There are no guidelines about font sizes for labels of in-vitro diagnostics. For the labels of Medicinal Products, characters of at least 7 points (or of a size where the lower case "x" is at least 1.4mm in height) with a space between the lines of at least 3 mm are recommended, according to "Guideline on the readability of the labelling and package leaflet of medicinal products for human use", Revision 1, 12 January 2009.
http://ec.europa.eu/health/files/eudralex/vol-2/c/2009_01_12_readability_guideline_final_en.pdf

4. The official **language(s)** in which the intended use is displayed, should be relevant to the region where the RDT product will be used.

What should be displayed:

1. Product name with sufficient details for the user to identify the product and its intended use, *e.g.*

- Commercial name of the RDT product
- "Malaria"
- targeted species and antigen(s)
- "Antigen" or "Ag"

Example: Commercial name, Malaria Pf/Pv (HRP2/pLDH) Antigen(RDT)

2. **Buffer** (Bottle)

3. **Product Code**

4. ***In vitro* diagnostic**

5. **Name of the legal manufacturer**

6. **Lot number**

7. **Expiration date**
Use the YYYY-MM format
The expiration date should not be earlier than the expiration date on the RDT box and device packaging

8. **Storage conditions** (symbols)

9. **Warnings or precautions** (symbols)
For instance: - do not use if package is damaged (symbol)
- hazard symbol if sodium azide concentration is ≥ 0.1% (symbol)

10. If the RDT product is CE-marked, the buffer bottle should have (and comply with) the **CE mark** too

Where should the labels be put

Proposal of how relevant information can be printed on the buffer bottle.

IVD 4 ℃ 30 ℃	
**Commercial name, Malaria Antigen Pf/Pan (HRP2/ pLDH) RDT**<br>**Product code: XXXXXX**	
**Buffer Bottle**	
LOT	XXXXXXX
	YYYY-MM
	Manufacturer Name
CE	

Example:

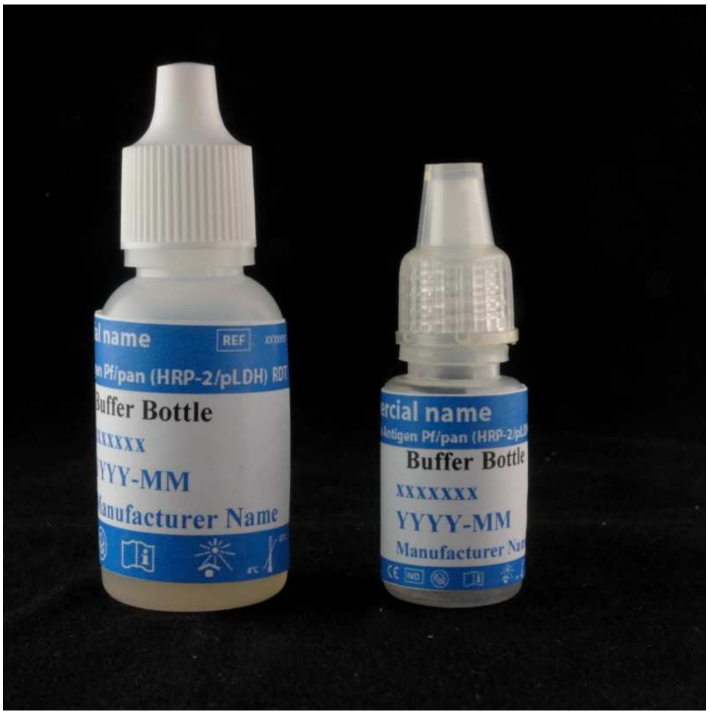

5. Labeling of the accessories

Definitions

Accessories of IVDs are articles specifically and explicitly intended by the manufacturer to be used together with a device to enable that device to be used in accordance with the intended purpose (ISO 18113-1, CE Directive 98/79). In this section, transfer devices, lancets, alcohol swabs and desiccant are intended.

General requirements and outlines:

1. **Labels:** Labels should be printed on the device or packaging as permanent printing or applied as water-resistant labels (with water resistant glue). Prints should be indelible and lasting the life span of the product. If it is not practicable to display the information on the device itself (e.g. lancets, transfer devices), some or all of the information may appear on the **packaging of multiple devices** (GHTF/SG1/N70/2011:5.0 and Annex 1.8.8.1 of EU Directive 98/79).

2.**Use of Symbols** is encouraged. Only Internationally recognized symbols (EN ISO 15223 – 2012) should be used.

3.**Labeling must be legible**: for example a type size of at least 7 points in font 'Times New Roman', not narrowed (or of a size where the lower case "x" is at least 1.4mm in height), with a space between lines of at least 3 mm, open letter type.

4. The official **language(s)** in which the intended use is displayed, should be relevant to the region where the RDT product will be used.

5. The table below lists the **Information** to be displayed on the different accessories or on their packaging.

**Information**	**Transfer device**	**Lancet**	**Alcohol swab**	**Desiccant**
Product name (of transfer device, lancet, alcohol swab, desiccant)	X	X	X	X
Intended use (sufficient to identify the device and its intended use: transfer device, antiseptic, desiccant…)	X	X	X	X
Name of the manufacturer	X	X	X	X
In case of alcohol swab: antiseptic, product and concentration (*e.g.* isopropyl alcohol70%)			X	
Product code	X	X	X	X
In case of transfer device other than inverted cup and loop: volume mark	X			
Lot number	X	X	X	X
EC and authorized representative (if applicable)	X	X	X	X
Indication of '*in vitro* diagnostic' use (for transfer device)	X			
Expiration date (YYYY-MM)		X	X	X
Numbers inside the outer package (if applicable) (symbol)	X	X	X	
Sample volume transferred	X			
Single use (symbol)	X	X	X	
Sterile (no need to mention "method of sterilization")		X	X	
Do not use if package is damaged (symbol)	X	X	X	
"Do not swallow/eat" and "harmful" (text)				X
Interpretation of color change (optional)				(X)
