# Supplementary material for: Harmonization of malaria rapid diagnostic tests: best practices in labelling including instructions for use
Source: Malar J. 2014 Dec 17;13:505. doi: 10.1186/1475-2875-13-505 (PMC4302121; doi:10.1186/1475-2875-13-505)
Supplement: Supplementary file 3 — Additional file 3: Generic template for Instructions for Use (IFU). (PDF 402 KB) [file 12936_2014_3663_MOESM3_ESM.pdf]

Additional file 3. Generic template for Instructions for Use (IFU)

The present document is a template for generic IFU of malaria rapid diagnostic tests.
It must be adapted to the specific product.
Words or terms that are definitely product-related and variable are already printed in blue.
Of course, this template can be adapted according to present or future characteristics of the concrete product. Instructions for the designer are printed in italics and put into text boxes.
The present document uses the safety-seal lancet and inverted cup as an example. Other combinations are possible.

General suggestions for the lay-out of the IFU in particular to the procedure:

- Provide IFU version number and date.

- Highlight changes with regard to the previous version.

- Text : make sure that the IFU is easily readable (e.g. Flesch-Kincaid grade < 6)

- *use type size of at least 9 points, as measured in font 'Times New Roman', not narrowed, with a space between lines of at least 3 mm and an open letter type*
- *short sentences and terms that are easy to understand*
- *use consistent terms and words throughout the IFU (see Terminology List)*
- *use active verb (imperative) rather than passive voice/"should"*
- *stress important information (capitals, italics, underline)*
- *turn any list into a bulleted or numbered list*
- *put "when" and "if" before "what" ("If the color indicator is red, discard the test").*
- *put the warning before the action step in the procedure*
- *make sure warnings are clearly indicated*
- *use one line per action*

For some references on readability and a readability calculator, refer to ***Annex 1****.*

- Figures: take the following into account:

- *use figures that are large enough so that they are easily visible*
- *drawings may be more informative than photographs*
- *the generic job aid for malaria RDTs published by WHO-FIND, 22 December 2009 provides clear drawings (see* ***Annex 2****)*
- *put figures at the left side, text at the right side*
- *refer to each figure in the text*
- *check that the figures match the real-life situation (device, transfer device, gloves, right-handed operator…).*

Table of contents

Product

- Product name
- Product Code
- Number of tests provided in the kit

Intended use

- Test principle
- Intended user
- Required specimen

Warnings and precautions

Materials

- Materials provided
- Materials required but not provided

Storage and stability

Procedure

- Before testing
- Test procedure
  - Capillary whole blood from finger prick
  - Venous whole blood from venipuncture
- Interpretation of the test result

Limitations of the test, causes of false-negative and false-positive results

- Limitations of Malaria RDTs
- False negative results
- False positive results
- Invalid tests and problems of background clearing

Performance specifications

Bibliography

Contact of Manufacturer

IFU version number and date of issue of the instructions for use

Symbol key

Product

- **Commercial name, Malaria Antigen Pf/Pan (HRP2/ pLDH) Rapid Diagnostic Test (RDT)**
- **Product code** xxxxxx

IVD

Intended use

This XXX test kit is an *in-vitro* diagnostic immunochromatographic assay for the qualitative detection of infection with *Plasmodium* parasites causing malaria in human whole blood specimens. It does not assess parasite densities.

It assists trained competent users
- in detecting *Plasmodium* infections
- to differentiate infection by *Plasmodium falciparum* from the non-*P. falciparum* species (*Plasmodium vivax, Plasmodium malariae, Plasmodium ovale*).

Note: Malaria RDTs can give positive results after successful anti-malarial treatment. Therefore, the XXX test kit is not recommended for monitoring response to anti-malarial treatment.

Test Principle

The following *Plasmodium* antigens are detected in this test:

- Histidine rich protein 2 specific for *P. falciparum* (Pf-HRP2)
- *Plasmodium* lactate dehydrogenase specific for *P. falciparum* (Pf-pLDH)
- *Plasmodium* lactate dehydrogenase specific for *P. vivax* (Pv-pLDH)
- *Plasmodium* lactate dehydrogenase common to all human *Plasmodium* species (pan-pLDH)
- Aldolase common to all human *Plasmodium* species

The cassette contains a test strip pre-coated with capture antibodies.
The sequence of events is as follows:
(1) Whole blood is applied to the specimen well (labelled well 1).
(2) Next, buffer is applied to the buffer well (labelled well 2).
(3) Migration of the blood/buffer mixture starts, towards the opposite end of the cassette.
(4) The blood-buffer mixture passes the conjugate pad, which contains detection antibodies targeting Pf-HRP2, Pf-pLDH, Pv-pLDH, pan-pLDH and/or aldolase antigens. These detection antibodies are conjugated to colloidal gold. If present in the specimen, *Plasmodium* target antigens bind to this detection antibody-conjugate.
(5) The antigen-antibody-conjugate complex migrates further and binds to the capture *Plasmodium*-specific antibodies present on the test line. These capture antibodies bind to another site (epitope) of the *Plasmodium* target antigens.
(6) The capture antibodies are applied on a narrow section of the test strip: as a result, the antibody-conjugate with the colloidal gold will be concentrated and become visible as a red colored line.
(7)The excess of the detection antibody-conjugate that was not bound by the *Plasmodium* target antigens and the capture antibodies moves further until it binds to a goat anti-mouse control antibody. There, the colloidal gold will create a red colored control line. The visualization of the control line indicates that the migration was successful. It does not confirm the presence of specimen.

The main ingredients are:

- Test strip:
    - Detection antibodies conjugated to colloidal gold :
        - Mouse monoclonal antibodies (IgG) specific to Pf-HRP2-gold Colloid
        - Mouse monoclonal antibodies (IgG) specific to pan-pLDH-gold Colloid
        - (any other combination)
    - Capture antibodies:
        - *Plasmodium falciparum* line: Mouse monoclonal antibodies (IgG) specific to Pf-HRP2
        - *Plasmodium* species (pan) line: Mouse monoclonal antibodies (IgG) specific to pan-pLDH
    - Control line : Goat anti-mouse polyclonal antibodies (IgG)
- Buffer vial:
    - Bovine serum albumin, Triton X-100, Sodium azide (0.095 %)

Intended user

- The test is intended to be performed by a trained user

Specimen required

- Capillary blood or venous blood with the following anticoagulant: EDTA, heparin, Oxalate or Citrate.
- Time between collection and analysis:
    - Capillary: immediately
    - Venous: immediately. If immediate testing is not possible, store the whole blood specimen at X-X °C for maximum XX hours.

! Do not use any other specimen than whole blood.

Warnings and Precautions

- For *in vitro* diagnostic use only.
- Read the instructions carefully before performing the test.
- Apply standard biosafety precautions for handling and disposal of potentially infective material.
    - Handle all specimens as potentially infectious.
    - Wear gloves while handling specimens and performing the test.
    - Avoid splashing and aerosol formation.
    - Clean up spills thoroughly using an appropriate disinfectant.
- The buffer contains 0.095% sodium azide as a preservative which may be toxic if ingested. When disposed of through a sink, flush with large quantities of water.
- Do not use any other buffer than the buffer supplied within this kit.
- Do not use the RDT kit beyond the expiration date.
- Do not use if the packaging is damaged.
- Do not use any other specimen than whole blood.
- Do not use if the product has been exposed to excessive heat or humidity.
- Perform the test immediately after opening of the cassette packaging.
- Do not re-use the test.

Materials

Materials provided

- XX cassette packagings , each containing:
    - 1 device
    - 1 desiccant
- X buffer bottles – XX ml
- XX specimen transfer devices (inverted cup) – x µl
- XX single-use safety-seal lancets
- XX alcohol swabs
- 1 Instructions for use

Materials required but not provided

- New pair of disposable gloves
- Pen
- Timer
- Extra lancets and alcohol swabs, if needed
- Sharps box
- Non-sharps disposal container
- Venipuncture blood collection materials and precision pipette plus tips (if whole blood is collected by venipuncture)

Test kit Storage and stability

- Store the kit between X-XX °C.
- Do not store the kit in the freezer.
- Protect the kit from humidity.
- The RDT kit has a shelf life of XX months from the date of manufacture. The test kit is stable until the expiration date marked on the RDT box and/or the packaging of individual components when stored as specified.

Procedure

See above for lay-out and style

Before testing:

1. Prepare all necessary materials :
   - When stored in the refrigerator, bring the kit components to room temperature minimum 30 minutes before use.
   - Prepare the materials:

Materials provided	Materials required but not provided
• Cassettes<br>• Buffer bottles<br>• Inverted cups<br>• Safety- seal lancets<br>• Alcohol swabs<br>• Instuctions for use	• New pair of disposable gloves<br>• Pen<br>• Timer<br>• Extra lancets and alcohol swabs, if needed<br>• Sharps box<br>• Non-sharps disposal container<br>• Venipuncture blood collection kit and precision pipette plus tips (if whole blood is collected by venipuncture)

2. Check the expiration date of the test.
   If expired, do not use it but take another test from an unexpired kit.
3. Check that the cassette packaging is not damaged.
   If damaged, discard the cassette packaging and use another test.
4. Open the cassette packaging and check the desiccant.
   If there is a humidity indicator and it shows saturation (color changed from orange to green), throw away the cassette and take another cassette packaging.
   If the color of the desiccant does not show a change, you can use the test.
   Throw away the desiccant in the non-sharps disposal container.
5. Take the cassette and place it on a horizontal surface.
   You see:
   - a result window (marked with C, pan, Pf)
   - a circle well marked “1” (for specimen)
   - a square well “2” (for buffer)
6. Write the patient name or patient identifier on the cassette.
7. Put on gloves. Use new gloves for each patient.
8. *Add if needed additional instructions on how to open the buffer bottle correctly – for instance, how to pierce the nozzle.*

! Perform the test immediately after opening of the cassette packaging.<br><br>! Do not re-use the test.

Test procedure *(see reference Generic RDT training manual in **Annex 2**)*

Capillary whole blood from finger prick

1. Wear gloves.
2. Choose a finger for the finger prick:
   - Do not choose a finger that is swollen, bruised or scarred.
   - Preferably choose the 3rd or 4th finger of the hand the patient does not use to write. Alternatively choose the heel or the earlobe for neonates.
3. Open the packaging of the alcohol swab. Take out the alcohol swab. Do not throw away the empty packaging (wrapper) but keep it aside.
4. Wipe the complete fingertip with the alcohol swab.
   Wait until the finger has completely dried (minimum 30 seconds).
5. Place the alcohol swab in the wrapper and set it aside (you will use it again to stop the bleeding after you collected the patient's blood).
6. Take the safety-seal lancet.
7. Detach the cap of the lancet.
   Puncture the side of the pulp (ball) of the finger with the lancet, perpendicular to the lines of the fingerprint.
   Dispose the lancet immediately into the sharps box.
8. Make sure a well-formed drop of blood is present on the tip of the finger.
9. If there is no well-formed drop of blood, repeat the finger prick.
   Use a new lancet and choose a different puncture site.
10. Take the inverted cup and collect 5 µl of blood by dipping the circular end of the inverted cup into the whole blood drop.
11. Place the circular end of the inverted cup in the circle well (marked "1") so that it touches the strip (pad at the bottom of the well)
    Press down lightly to transfer the whole blood to the strip.
    Put the used inverted cup into the non-sharps disposal container.
12. Take the alcohol swab you put aside (step 5).
    Ask the patient to press it to the finger prick to stop the bleeding.
    After use, put the alcohol swab into the non-sharps disposal container.
13. Take the buffer bottle.
    Hold the open buffer bottle vertically above the square well (marked "2").
    Squeeze the buffer bottle gently and apply exactly X drops into the square well (marked "2").

> ! Avoid the tip or center of the finger.
>
> ! Avoid the side of the finger.

> ! Do not use any other buffer than the buffer supplied within this kit.

> ! Hold the buffer bottle vertically – this ensures that the drops contain the correct volume of buffer.

14. Remove your gloves and discard them into the non-sharps disposal container.
15. Write the time on the cassette or set a countdown timer to the required reading time.
16. Read test results after a minimum of xx minutes but no later than xx minutes.
    Use a good light source when reading the test results.

> ! Do not read results after xx minutes.

Venous whole blood from venipuncture

1. Wear gloves.
2. Collect blood by standard venipuncture procedure into a tube containing the correct anticoagulant (EDTA, heparin, Oxalate or Citrate).
3. Mix the tube gently.
4. Transfer 5 µl of whole blood in the circle well (marked "1") of the cassette using a precision pipette.

5. Perform steps 12 - 16 of the previous section (“Capillary whole blood from finger prick”)

Interpretation of the test result

1. After xx but no later than xx minutes: compare the test lines with the presentation in the table below.
2. Where possible, have the results confirmed by a second reader within this time frame.
3. Line intensities may vary from faint to strong intensity.
   Consider also a faint test line as a positive result.
4. Record the test results as noted in the table below .
   Consult the national guidelines for malaria case management to complement the table below.

**Lines that you see**	**Picture/Drawing**	**Record the following result**<br>**Take the following action**
**NO line at ‘C’**<br>(= control)	*Put figures of all possible line combinations*	Invalid<br>Take a new cassette packaging and repeat the test!
**Line at ‘C’** and<br>**NO** other line	*Put figures of all possible line combinations*	Negative
Line at ‘**C**’ AND at ‘**Pf**’	*Put figures of all possible line combinations*	Positive for *Plasmodium falciparum*
Line at ‘**C**’, at ‘**Pf**’ AND at ‘**pan**’	*Put figures of all possible line combinations*	Positive for Plasmodium falciparum (or rarely, a mixed infection with *P. vivax, P. ovale* and/or *P. malariae)*
Line at ‘**C**’ AND at ‘**pan**’	*Put figures of all possible line combinations*	Positive for non-*falciparum* malaria: *P. vivax, P. ovale* or *P. malariae* (or, rarely, a mixed infection with these species)
Other line combinations	*Put figures of all possible line combinations*	*Write down the result*

Note: the XXX test kit does not differentiate between *P. vivax*, *P. ovale* and *P. malariae*

Limitations of the product, causes of false-negative and false-positive results

All malaria RDTs have limitations in common.<br>*They may be related to the RDT, the end-user and the conditions during transport and storage.*<br>*They may occur despite correct storage and procedure and are related to:*<br>• *the general design of the RDT (detection limit, prozone, no quantification)*<br>• *the antigen (HRP-2 deletions, HRP-2 persistence after treatment)*<br>• *the operator (overlooking faint test lines)*<br>• *the species (in general: sensitivity for P. falciparum > P. vivax > P. ovale/malariae)*<br>*We listed the limitations below – unless they do not apply for the RDT product under consideration, they should be mentioned.*<br><br>*See also reference "Universal access to malaria diagnostic testing: an operational manual. World Health Organization 2011"*

Malaria RDT have limitations

They may be the cause of

- false-negative results (no test lines but the patient has malaria)
- false-positive results (test lines visible but the patient does not have malaria)
- invalid test result (no control line and/or incomplete clearing of background)

Sensitivity for detecting malaria is lower in the case of *P. ovale* and *P. malariae*.

False negative results can occur in the following conditions:

- very low antigen concentrations/parasite densities, for instance < 100 parasites/µl. Note that most clinical cases have higher parasite densities.
- very high parasite densities (very exceptional, prozone or high-hook effect) for the HRP-2 antigen
- deletions in the HRP-2 gene resulting in no production of the HRP-2 antigen (of relevance only for mRDTs that detect this antigen, and only significantly present in the Peruvian Amazon)

False positive results can occur – amongst others- in the following conditions:

- rheumatoid factors, antinuclear antibodies
- viral infection (such as hepatitis B or hepatitis C, dengue)
- parasitic infection (such as schistosomiasis and trypanosomiasis)

Invalid tests and problems of background clearing may occur:

- In lipaemic and icteric specimens

<u>Note</u>: The presence of the control line only means that migration of the test occurred. It does not guarantee that:

- the correct specimen has been used
- the specimen has been applied correctly
- the specimen and test have been correctly stored
- the test procedure was followed correctly

Performance specifications

<u>Recommendations for diagnostic performance specifications :</u>

- *State at least the following specifications and information:*

1. Analytical sensitivity (detection limit)
2. Analytical specificity (rheumatoid factor, antinuclear antibody, other infections and influence of lipemic/icteric/hemolyzed specimens)
3. Diagnostic sensitivity
4. Diagnostic specificity
5. Repeatability (test-related, laboratory conditions)
6. Reproducibility (operator-related, field conditions)

- *Give enough detail and oversight:*
  - *the numbers of specimens used (and if applicable, confidence intervals)*
  - *the different specifications for P. falciparum, P.vivax, P. ovale and P. malariae*
  - *type of study and setting, geographic place, study period and population (laboratory study on stored specimens, clinical study, field study, …)*
  - *parasite* densities *and reference methods when appropriate (for instance in the case of diagnostic sensitivity)*
  - *present results in a clear way (e.g. table)*
  - *refer to type of study (in-house study, external study, study report or published in scientific literature)/include a bibliography/reference list*

Bibliogaphy

Recommendations for bibliography:
Select relevant publications in a practical and product-oriented way.
*In **Annex 3** we give some references for relevant topics.*

Product-related publications

- Test kit evaluations (product related studies)

General publications

- Biosafety and Sampling
- WHO Product Testing rounds
- Description of problems on RDT implementation, end-user errors (included prozone, buffer substitution, false positive,etc.)

Contact of manufacturer

Name of the legal manufacturer
Physical address of the manufacturing site
Contact for technical assistance (telephone/fax number, email address)

If applicable CE and the authorized representative in the European Community EC REP

Version number of IFU and date of issue: XXXXX, YYYY/MM/DD

Symbol key

Recommendations for Symbol key:

- *Only use internationally recognized symbols.*
- *In **Annex 4** we give an example of a symbol key*

Annex 1: References for readability

The following websites explain how to assess and calculate readability – the tool is primarily made for English texts.

http://www.online-utility.org/english/readability_test_and_improve.jsp

http://www.mang.canterbury.ac.nz/writing_guide/writing/flesch.shtml

Readility can also be assessed in a Microscoft Word-document:

1. Click the File tab, and then click Options.

2. Click Proofing.

3. Under "When correcting spelling and grammar in Word", make sure the "Check grammar with spelling check" box is selected.

4. Select "Show readability statistics" and click on "OK"

After you enable this feature, open a file that you want to check, and check the spelling. When Outlook or Word finishes checking the spelling and grammar, it displays information about the reading level of the document.

Annex 2:
Generic and product specific job aids for Pf –only and combination RDT

Refer to the following website :

Generic : http://www.wpro.who.int/malaria/sites/rdt/home.html

Product specific : http://www.finddiagnostics.org/programs/malaria-afs/malaria/rdt-job-aids/

Generic RDT training manual:

How to use a rapid diagnostic test (RDT): a guide for training at a village and clinic level 2009. The USAID Quality Assurance Project (QAP),Unversiversity Research Co., LLC, and the World Health Organization (WHO), Bethesda, MD, and Geneva

http://www.wpro.who.int/malaria/sites/rdt/using_rdts/training/main.html

Universal access to malaria diagnostic testing: an operational manual. World Health Organization 2011

http://www.who.int/malaria/publications/atoz/9789241502092/en/

Annex 3: Example of bibliography

Product-related publications

- Test evaluations (product related study)

General publications

- Biosafety and Sampling

1. Clinical and Laboratory Standards Institute. Procedures and devices for the collection of diagnostic capillary blood specimens; approved standard, fifth edition. CLSI H04-A6, Vol. 28, No. 25, 2008.
2. Clinical and Laboratory Standards Institute. Procedures for the collection of diagnostic blood specimens by venipuncture; approved standard, sixth edition. CLSI H03-A6, Vol. 27, No. 26, 2007
3. World Health Organization: Laboratory biosafety manual, third edition. Geneva: WHO; 2004. http://www.who.int/csr/resources/publications/biosafety/Biosafety7.pdf

- WHO Product Testing rounds

1. World Health Organization: Developing and testing a generic job aid for malaria rapid diagnostic tests (RDTs). Geneva: WHO; 2004. http://www.wpro.who.int/malaria/NR/rdonlyres/C717F47F-DA04-469E-BEA3-C1176F720257/0/Developing_and_testing_an_RDT_Job_Aid.pdf
2. World Health Organization: Good practices for selecting and procuring rapid diagnostic tests for malaria. Geneva: WHO; 2011. http://whqlibdoc.who.int/publications/2011/9789241501125_eng.pdf
3. World Health Organization: Guidelines for the treatment of malaria. Second edition. Geneva: WHO; 2009. Geneva: WHO; 2009. http://whqlibdoc.who.int/publications/2010/9789241547925_eng.pdf
4. World Health Organization: Malaria Rapid Diagnostic Test Performance; Results of WHO product testing of malaria RDTs: Round 4 (2012). Geneva: WHO; 2012. http://www.finddiagnostics.org/resource-centre/reports_brochures/malaria-diagnostic-test-report.html
5. World Health Organization: Malaria RDT Job-Aids and Training Manuals http://www.wpro.who.int/malaria/sites/rdt/using_rdts/training/index.html
6. World Health Organization: Management of severe malaria – A practical handbook. Third edition. Geneva: WHO; 2013. http://apps.who.int/iris/bitstream/10665/79317/1/9789241548526_eng.pdf
7. World Health Organization: Transporting, storing and handling malaria rapid diagnostic tests at central and peripheral storage facilities. Geneva: WHO; 2009. http://www.who.int/malaria/publications/atoz/malaria_rdt_central_2009.pdf
8. World Health Organization: Universal access to malaria diagnostic testing. An operational manual. Geneva: WHO; 2011. http://whqlibdoc.who.int/publications/2011/9789241502092_eng.pdf

- Description of problems on RDT implementation, end-user errors (included prozone, buffer substitution, false positive,…)

1. Gamboa D, Ho M, Bendezu J, Torres K, Chiodini P, Barnwell J, Incardona S, Perkins M, Bell D, McCarthy J, Cheng Q: A large proportion of P. falciparum isolates in the Amazon region of Peru lack pfhrp2 and pfhrp3: implications for malaria rapid diagnostic tests. *PLoS One* 2010, 5:e8091. http://www.plosone.org/article/info%3Adoi%2F10.1371%2Fjournal.pone.0008091
2. Gillet P, Scheirlinck A, Stokx J, De Weggeleire A, Chauque H, Canhanga O, Tadeu B, Mosse C, Tiago A, Mabunda S, Bruggeman C, Bottieau E, Jacobs J: Prozone in malaria rapid diagnostics tests: how many cases are missed? *Malar J* 2011, 10:166. http://www.malariajournal.com/content/10/1/166
3. Gillet P, Mori M, Van Den Ende J, Jacobs J: Buffer substitution in malaria rapid diagnostic tests causes false-positive results. *Malar J* 2010, 9:215 http://www.malariajournal.com/content/9/1/215
4. Maltha J, Gillet P, Cnops L, Van Den Ende J, Van Esbroeck M, Jacobs J: Malaria rapid diagnostic tests: Plasmodium falciparum infections with high parasite densities may generate false positive Plasmodium vivax pLDH lines. *Malar J* 2010, 9:198. http://www.malariajournal.com/content/9/1/198
5. Maltha J., Gillet P., Jacobs J. REVIEW: Malaria rapid diagnostic tests in endemic settings. *Clin Microbiol Infect 2013; 19: 399–407.* http://onlinelibrary.wiley.com/doi/10.1111/1469-0691.12151/pdf
6. Maltha J., Gillet P., Jacobs J. REVIEW: Malaria rapid diagnostic tests in travel medicine. *Clin Microbiol Infect* 2013; 19: 408–415. http://onlinelibrary.wiley.com/doi/10.1111/1469-0691.12152/pdf

Annex 4: Example of symbol legend

Symbol	Explanation	Symbol	Explanation
IVD	*In vitro* diagnostic medical device	REF	Product code
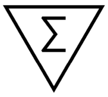	Content sufficient for < n > tests	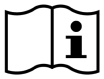	Consult instructions for use
LOT	Lot number	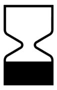	Use by YYYY-MM-(DD)
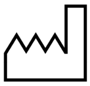	Date of manufacture YYYY-MM-(DD)	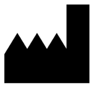	Manufacturer
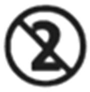	Do not reuse	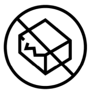	Do not use if packaging is damaged
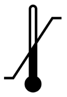	Temperature limitation	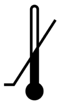	Lower limit of temperature
STERILE	Sterile	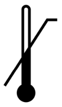	Upper limit of temperature
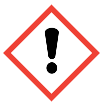	Irritant	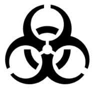	Biological risk
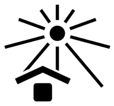	Keep away from sunlight	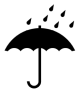	Keep dry
EC REP	Authorized representative in the European Community	CE	European Health & Safety Product Label
